# In Situ Mass Spectrometry Imaging and Ex Vivo Characterization of Renal Crystalline Deposits Induced in Multiple Preclinical Drug Toxicology Studies

**DOI:** 10.1371/journal.pone.0047353

**Published:** 2012-10-23

**Authors:** Anna Nilsson, Benita Forngren, Sivert Bjurström, Richard J. A. Goodwin, Elisa Basmaci, Ingela Gustafsson, Anita Annas, Dennis Hellgren, Alexander Svanhagen, Per E. Andrén, Johan Lindberg

**Affiliations:** 1 Medical Mass Spectrometry, Department of Pharmaceutical Biosciences, Uppsala University, Uppsala, Sweden; 2 Safety Assessment, AstraZeneca R&D, Södertälje, Sweden; 3 Global Distribution Imaging, DMPK IM, AstraZeneca R&D, Södertälje, Sweden; Biological Research Centre of the Hungarian Academy of Sciences, Hungary

## Abstract

Drug toxicity observed in animal studies during drug development accounts for the discontinuation of many drug candidates, with the kidney being a major site of tissue damage. Extensive investigations are often required to reveal the mechanisms underlying such toxicological events and in the case of crystalline deposits the chemical composition can be problematic to determine. In the present study, we have used mass spectrometry imaging combined with a set of advanced analytical techniques to characterize such crystalline deposits *in situ.* Two potential microsomal prostaglandin E synthase 1 inhibitors, with similar chemical structure, were administered to rats over a seven day period. This resulted in kidney damage with marked tubular degeneration/regeneration and crystal deposits within the tissue that was detected by histopathology. Results from direct tissue section analysis by matrix-assisted laser desorption ionization mass spectrometry imaging were combined with data obtained following manual crystal dissection analyzed by liquid chromatography mass spectrometry and nuclear magnetic resonance spectroscopy. The chemical composition of the crystal deposits was successfully identified as a common metabolite, bisulphonamide, of the two drug candidates. In addition, an un-targeted analysis revealed molecular changes in the kidney that were specifically associated with the area of the tissue defined as pathologically damaged. In the presented study, we show the usefulness of combining mass spectrometry imaging with an array of powerful analytical tools to solve complex toxicological problems occurring during drug development.

## Introduction

Understanding the circumstances of toxicological events arising during the development of new candidate drugs is crucial during pharmaceutical research, often requiring thorough investigations to understand toxicological findings. Mechanistic information is then able to feedback and allows candidate drug refinement and redevelopment [1], [2]. In preclinical studies the bio-distribution of a drug is routinely measured by quantitative whole body autoradiography (QWBA) as well as by scrutinizing *in vitro* plasma drug levels and drug-protein binding levels [3]. While such assays are invaluable they are limited in their scope, for example providing quantitative data on compound spatial distribution but failing to report on metabolite accumulations or biomarker changes. Hence, when such studies are undertaken during preclinical safety investigations, the data collected do not always correlate with later toxicological findings [3], [4].

Drug-induced kidney injury is a major toxicological side effect that is often detected during drug development [1], [5], [6] and requires extensive study to enable a compound specific understanding to be obtained. A better understanding of systemic renal toxicological damage could allow identification of possible biomarkers for early detection of kidney damage. A common finding of nephrotoxicity is accumulation of crystalline deposits within the kidney, the composition of which are often difficult to determine. While compound properties are investigated during ADME (adsorption, distribution, metabolism, and elimination) and in toxicology studies across many different animal models, the mechanisms underlying crystal formation and pathologic outcomes are not always clear. Therefore, once toxicological events are identified, a raft of established and recently evolved bioanalytical technologies (complementary to the standard assays) are needed to define, identify and confirm the cause and effect of the toxicological incidents. A full assessment of renal crystalline deposit can be separated into two stages. Firstly, there is a targeted investigation which aims to determine the identification of crystalline formations detected by histopathology. Following their identification and confirmation, there can be on-tissue monitoring of the distribution of the identified compounds. Secondly, there is an untargeted evaluation of tissue samples, with the aim of identifying biomolecular changes that cannot be detected by histopathology.

Both assessment stages, targeted and untargeted, require the use of a number of complementary technologies to provide cross validation of the results obtained. High performance liquid chromatography coupled to mass spectrometry (LC-MS), for example, is a very powerful and extensively used technology for the determination of drug and metabolite abundances in tissue samples [7]–[9]. However, it requires tissue extracts for analysis and therefore there is loss of all spatial information. Some spatial information can be retained through the use of sample collection techniques such as laser capture micro-dissection which enables the collection of single cells (or populations of cells) from tissue sections [10]–[12]. However, as a laser beam is used to selectively cut out sample areas the heat introduced may compromise the sample. Further complications can arise with sample processing and analysis of such minute samples, with increased risk of sample loss or contamination. A simpler selective approach is manual dissection and collection of the crystal structures. Such a method removes the risk of heat-damage, but there still remains the complication of subsequent sample processing and analysis. These methods can be very sensitive during the identification of a substance, but are limited in their ability to provide any indication of relative abundance of the compound across a tissue sample or provide *in situ* identification or information on co-localization of additional underlying components of the sample. Therefore, technologies that allow the direct analysis of a sample with no, or minimal, processing are proving to be very beneficial when dealing with such samples. High resolution magic angle spinning spectroscopy (HRMAS) nuclear magnetic resonance (NMR) is an example of a technology that provides a potential untargeted approach to analyzing unprocessed tissue sample sections [13] that complements LC-MS analysis of tissue section extracts.

The recently developed technique of matrix assisted laser desorption ionization (MALDI) mass spectrometry imaging (MSI) is a powerful label-free technology that enables the multiplexed and simultaneous analysis of a variety of different endogenous and exogenous molecules directly from the surface of tissue sections. This technique maintains spatial information and is therefore suited to both targeted and untargeted analysis of tissue samples [14]–[16]. MALDI MSI enables the direct measurement of ion distributions and abundances of drugs, metabolites, lipids, peptides and small proteins [17]–[22]. The technique, unlike some established technologies such as autoradiography, enables the simultaneous monitoring of parent compounds and metabolites without requiring either to be labeled [16]. Several recent reports and reviews demonstrate the feasibility of MALDI MSI in pharmaceutical research and describe in detail the sample preparation and processing required [16], [21], [23]–[25]. In relation to toxicological research applications, MALDI MSI has been used to identify accumulation of an active drug in areas where microcrystalline deposits were observed following administration of a pro-drug [26].

We present here the findings from a rat renal toxicological study involving the administration of two potential microsomal prostaglandin E synthase 1 (mPGES-1) inhibitors, with similar chemical structure, over a seven day period. Both drug candidate dosing regimens resulted in kidney damage with marked tubular degeneration/regeneration and crystal deposits within the tissue, detected by histopathology analysis. Both targeted and untargeted experiments were performed that identified the crystals as a common metabolite of the administered drugs. Using MALDI MSI it was also possible to measure the *in situ* distribution of the parent compounds while simultaneously revealing underlying biomolecular changes that could not be observed by histopathology. The approach we have undertaken combines histopathology, manual crystal dissection, LC-MS, NMR and MALDI MSI analysis to provide a robust suite of analytical techniques that can readily be applied by researchers investigating other toxicological events.

## Materials and Methods

A brief summary of experimental procedures is given here. Detailed information is provided in Text S1.

### Chemicals

**Figure 1:**
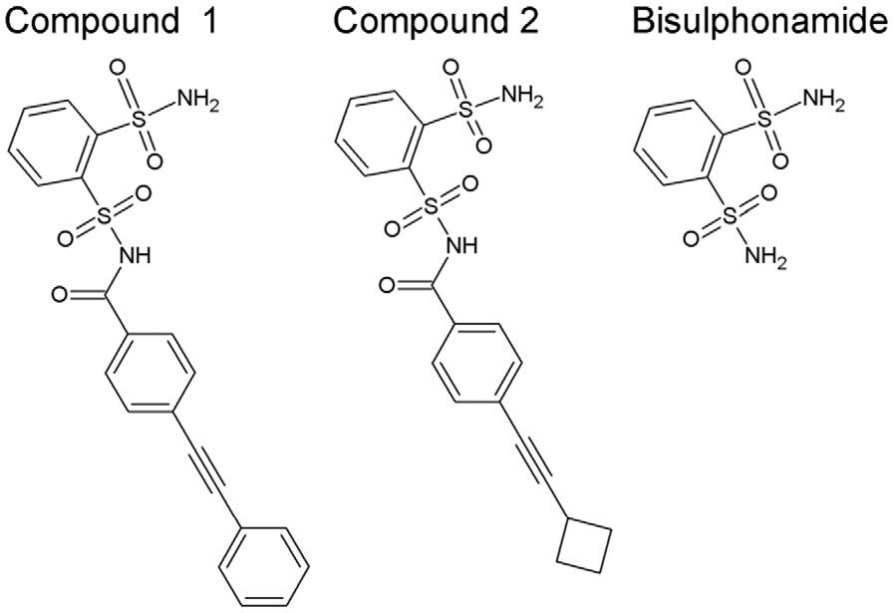
The structures of the two administered compounds (compound 1; 4-(phenylethynyl)-N-[(2-sulfamoylphenyl)sulfonyl]benzamide, compound 2; 4-(cyclobutylethynyl)-N-[(2-sulfamoylphenyl)sulfonyl] benzamide), and their common metabolite bisulphonamide (benzene-1,2-disulphonamide).

The drugs (compound 1; 4-(phenylethynyl)-N-[(2-sulfamoylphenyl)sulfonyl]benzamide; compound 2; 4-(cyclobutylethynyl)-N-[(2-sulfamoylphenyl)sulfonyl] benzamide; Figure 1), bisulphonamide (Figure 1), polyethylene glycol 400 (PEG400), HydroxyPropyl-β-cyclodextrin (HPβCD) hydroxypropyl methylcellulose (HPMC), and meglumine were acquired from AstraZeneca R&D (Sweden). DMSO-d6 (99.9%) was obtained from Cambridge Isotope Laboratories, Inc (USA) and acetonitrile of LC-MS grade was from Scharlau (Fisher Scientific, USA). All of the other agents and materials were of highest grade available.

### Tissue Samples

The study was conducted in accordance with the European Communities Council directive of 24 November 1986 (86/609/EEC) and was approved by the local ethical committee (Stockholm South Ethical Committees on Animal Experiments, Dnr S60/09 and S32/10). Female Wistar Hannover Galas rats, approximately 10 weeks old, weighing 170–250 g (Taconic M&B A/S, Denmark) were used. Two discovery compounds were administered by oral gavage. Compound 1 was given at dose levels of 0 and 440 mg/kg/day as a solution in 20% (v/v) polyethylene glycol (PEG400) in 0.2 M meglumine. Compound 2 was administered at a dose level of 0 and 1200 mg/kg/day formulated in 15% (w/v) HydroxyPropyl-β-cyclodextrin (HPβCD) in 0.2 M meglumine. All animals were dosed once daily for seven days and killed for scheduled necropsy on the day after the final dose. The kidneys were rapidly removed and divided into halves. The kidney halves intended for histological examination were fixed in a 4% buffered formalin solution (Solveco AB, Stockholm, Sweden), embedded in paraffin, sectioned and stained with hematoxylin and eosin (H&E). The other kidney halves were snap frozen in a mixture of isopentane and dry-ice and then kept at −80°C until analysis. These samples were sectioned for MALDI MSI, NMR HRMAS, and kidney extract preparation.

### Crystal Analysis

Crystals detected after histological examination were manually dissected from kidney tissue sections under microscope using a needle and were dissolved in 10 µL NMR solvent (DMSO-d6). 1 µL of this sample was used for LC-MS analysis and the remaining sample was used for NMR analysis.

### MALDI MS Imaging

Three different MALDI matrices, alpha-cyano-4-hydroxycinnamic acid (CHCA, 10 mg/mL), sinapinic acid (SA, 10 mg/mL), and 2,5-dihydroxybenzoic acid (DHB, 60 mg/mL) in 50∶50∶0.1 ACN/H_2_O/TFA were tested to optimize ionization conditions for the administered compounds. Prior to matrix application, drug standards (compound 1, 2 and bisulphonamide) were manually spotted onto control kidney tissue. The matrix was applied using an automatic spraying device (ImagePrep, Bruker Daltonics, Germany). The coated tissue sections were dried in the desiccator for approximately 20 min before MSI analysis. MALDI MSI experiments were carried out using the UltraFlex II MALDI-TOF/TOF MS (Bruker Daltonics) or the Synapt G2 HDMS system (MALDI Q-TOF MS, Waters Corp., UK). The mass spectrometer parameters were as per the manufacturers recommendations adjusted for optimal imaging performance (detailed information can be found in Text S1). The data acquired on the Ultraflex II was analyzed and normalized using FlexImaging 2.0. The tissue section from each individual animal was defined as the regions of interest using the optical scanned image. Ion distribution images of masses where average abundance was different between tissues from control and dosed animals were extracted with a mass filter precision of ±0.1 Da. Following acquisition on the Synapt G2, raw data was converted into Analyze file format using MALDI imaging converter (Waters) and normalized by total ion current using an in-house written script. Subsequent data analysis was performed using BioMap (Novartis, Switzerland) and Origin 8 (Origin Lab, USA). Ion distribution images were extracted using a mass filter of approximately ±0.015 Da depending on peak shape. The mass filter was kept constant for a specific mass on different tissues. All negative ion mode data was acquired on the Synapt G2.

### NMR Analysis

The NMR spectroscopic measurements were made on a Bruker 600 MHz instrument equipped with a sample changer (Bruker BioSpin, Germany). The ^1^H-NMR spectra of the DMSO-d6 soaked kidney tissue were acquired with a HRMAS probe using the standard Bruker cpmg presat puls program with suppression of the residual water resonance. The DMSO-d6 phase obtained upon centrifugation and the DMSO-d6 dissolved manually isolated crystals were analyzed with a 1 mm TXI probe using the standard Bruker noesygppr1d pulse sequence with suppression of the residual water. All data acquisition and processing was done with Topspin2.1 (Bruker BioSpin, Germany).

### LC-MS Analysis

The LC-MS analysis was performed using an Ultra Performance Liquid Chromatography (UPLC)/Q-TOF MS (Q-TOF Premier, Waters Corp.). Kidney extracts were analyzed in both positive and negative ionization modes using two different chromatographic systems; a) A HSS T3, 2.1×100 mm, 1.8 µm column (Waters), with a flow rate of 0.6 mL/min. Mobile phase A was 0.1% formic acid in water and mobile phase B was 0.1% formic acid in acetonitrile. The gradient was 1–20% B 0–5.5 min, 20–90% B 5.5–9 min, 90% B 9–13 min. b) A BEH Amide 2.1×100 mm, 1.7 µm column (Waters), with a flow rate of 0.3 mL/min. Mobile phase A acetonitrile and mobile phase B was 5 mM formic acid in water. The gradient was 10–50% B 0–5 min. A volume of 5 µL of sample was injected for all analyses. The mass spectrometer was operated in full scan mode (*m/z* 50–1000), with the collision cell held at 5 eV in MS mode. MS/MS experiments were performed at collision energies of 5 eV, 10 eV, and 20 eV to obtain MS/MS spectra at different energies. For profiling analysis of kidney extracts, an in-house designed software (TracMass [27]) was used to extract the most distinct differences between the samples.

## Results and Discussion

In the present study, both targeted and untargeted analysis strategies were performed during the examination of preclinical toxicological crystal formations detected in rat kidneys following a one week administration of two candidate drugs acting as mPGES inhibitors intended for the treatment of inflammatory diseases. Directed by histopathology, the crystals were manually dissected and analyzed by a combination of LC-MS and NMR. In addition, the relative abundance distribution of the compound identified in the crystals was mapped directly on tissue sections by MALDI MSI. In parallel, an untargeted analysis of molecular changes between damaged kidneys and vehicle controls was performed, also using NMR, LC-MS and MALDI MSI analysis.

### Ex vivo Crystal Analysis

**Figure 2:**
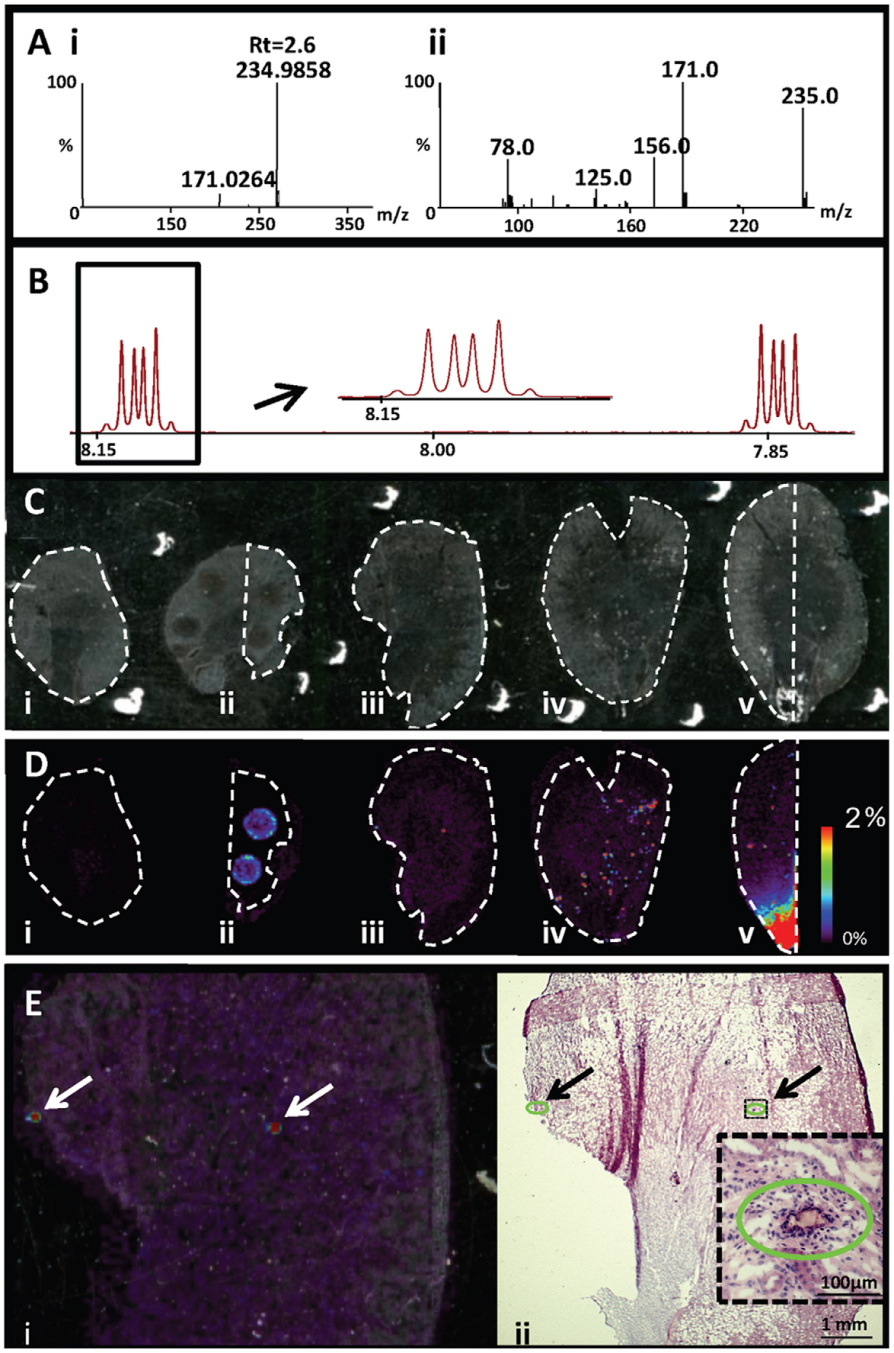
Identification and mapping of renal crystalline deposits. A) i; Mass spectrum of crystals isolated from kidney tissue analyzed by LC-MS. Retention time and mass-to-charge ratio matches that of bisulphonamide standard. ii; MS/MS spectrum of 234.98 observed in the crystal isolate. The fragmentation pattern matches that of bisulphonamide standard. B)^ 1^H-NMR spectra of the aromatic proton region of crystals from kidney tissue dissolved in DMSO. C) Optical images of analyzed tissue sections. i) vehicle control, ii) bisulphonamide standard on vehicle control, iii) compound 1 dosed tissue with a low crystal load, iv) compound 1 dosed tissue with a high density of crystals, v) compound 2 dosed tissue with a high crystal load. All samples were coated with sinapinic acid and analyzed in negative mode on the G2 Synapt. The data was normalized by total ion count. D) Ion distribution of bisulphonamide (m/z 235) on the tissue sections in panel C. The color intensity scale is adjusted to 2% of the maximum intensity on tissue v in order to visualize the distribution patterns on all tissues using the same color intensity scale. This means that pixels on the tissues in panel D above this value appear saturated. Data was acquired at a spatial resolution of 100 µm. E) Ion distribution of bisulphonamide overlaid on the tissue sections from the animal with a low crystal load following administration of compound 1. Left; ion distribution image of *m/z* 235 overlaid on scanned image of tissue section. Crystals are marked with arrows. Right; scanned image following H&E staining of the same tissue with example of a crystalline deposit in the kidney surrounded by a slight mononuclear cell reaction. Crystals are marked with arrows and circled in green. The size of the majority of the crystals ranged between 50 and 100 µm.

Indications of renal damage was initially detected when analysis of blood samples from animals receiving both drug dosed treatments showed increased plasma levels of urea, creatinine and potassium. Postmortem examination of kidneys revealed a pale discoloration and increased weight of the drug treated rat kidneys compared to controls (data not shown). Histopathological examination of kidney tissue sections showed marked tubular degeneration/regeneration, multiple accumulations of neutrophils and macrophages surrounding crystalline material within tubules in the cortex and medulla, inflammation, hemorrhage, transitional cell hyperplasia and free crystals in the renal pelvis. The crystal sizes varied but were typically in the range between 50–200 µm (Figure 2). Identification of crystal content, for both drug treatment regimes, was performed by LC-MS and NMR analysis (Figure 2). LC-MS was used to provide information on exact compound mass and substructure information through MS/MS data, while the ^1^H-NMR provided complementary structural information such as stereochemistry and number of protons in the analyte as well as an overall estimation of the purity of the crystals. Individual crystals were collected by manual dissection to prevent possible contamination that could occur during laser micro-dissection/micro-capture. Following the collection of crystals, LC-MS analysis identified bisulphonamide (*m/z* 235) to be the main component of all the crystals (Figure 2 Ai). Retention time, mass spectra and the MS/MS spectra (Figure 2 Aii) of the crystal content matched with that of synthetic bisulphonamide. No other significant analyte peaks were detected in the LC-MS analysis. Crystal composition was further confirmed by ^1^H NMR analysis. Again, the only major component of all samples of the dissected crystals was bisulphonamide. The characteristic pattern of two multiplets in the aromatic proton region (Figure 2B) could be determined as originating from bisulphonamide by the addition of this substance to one of the samples. No other analytes were detected. Crystal deposits displaying the same morphology were observed post-administration of the two structurally similar drugs. We can therefore conclude that their common metabolite bisulphonamide (Figure 1) is precipitating in the tissue and forming the crystal deposits. Bisulphonamide has structural resemblance to the group of structures called sulpha drugs, which are antibacterial sulfonamides. These compounds are known to cause crystal-induced acute renal failure [28].

### In situ Crystal Analysis

The ex vivo analysis of crystals enables sensitive identification of the chemical composition of the crystals, however, all spatial distribution data is lost (excluding knowledge of approximate location crystals were collected) and no information is obtained about the distribution of the parent drug or other molecular non-crystalline distributions within the tissue sections. MALDI MSI analysis was performed to provide a better understanding of the abundance and distribution of the bisulphonamide crystals (with the aim of directly relating MSI data to histopathology images) as well as mapping the parent drug distributions across kidney tissue sections. As MALDI MSI is a multiplex analysis with data collected simultaneously across a broad mass range, other underlying molecular changes can be detected while measuring the parent drug and bisulphonamide distributions.

### MALDI MSI Analysis of Bisulphonamide Crystal Distributions

Selection of MALDI MSI matrix and analysis conditions were optimized for both candidate drugs and bisulphonamide. Detection efficiency was determined to be greatest in negative mode using sinapinic acid (SA) as matrix on the Synapt G2 HDMS System. Bisulphonamide detection was increased ten times using SA compared to CHCA, while the detection of compound 1 and compound 2 was comparable using SA and CHCA. Therefore, following an application of SA matrix to kidney tissue sections, analysis was performed in negative ionization mode to map the distribution of crystals. To be able to confirm the identification of the crystals, bisulphonamide standard was manually spotted on to vehicle control tissue and analyzed alongside the drug treated sections. Both MALDI MSI and MS/MS imaging analysis were performed. MSI mapping of the distribution of *m/z* 235 shows that the bisulphonamide standard can clearly be detected where manually deposited (Figure 2D ii). However, more significantly, on drug treated kidney sections crystal-like patterns are detected (Figure 2D iii–v). MS/MS fragmentation of *m/z* 235 of both the bisulphonamide standard and for the ion on the crystals produces the same fragmentation pattern (Figure S1), verifying that the compound detected in the crystals is bisulphonamide and confirming the LC-MS and NMR analysis described earlier. The highest abundance of bisulphonamide was observed in the pelvic area in the kidney section from the animal administered compound 2 (Figure 2D v). On the tissue section from the animal given compound 1, where the histology examination showed a low load of crystals, only two raster positions displayed an intense bisulphonamide signal (Figure 2D iii, 2E). On the second kidney section from another animal given the same compound, the ion distribution of bisulphonamide was scattered across more of the entire tissue section confirming the histology report of numerous crystals being present. To match the distribution patterns of bisulphonamide observed by MSI to the crystal localization observed by histopathology, sections that had been analyzed by MSI were washed to remove the MALDI matrix prior to being H&E stained and imaged. The optical image was then aligned to the MALDI MSI distribution data for *m/z* 235 and showed excellent correlation between the locations of the crystals in all investigated samples. An example of the correlation between MSI and histopathology crystal distributions is presented in Figure 2E. It should be noted that spectra from positions where bisulphonamide was detected were analyzed for any co-localized *m/z* compounds that also could be part of the crystal deposits, however, no such *m/z* ratios were found in the investigated mass range. While the identification of the crystals was previously determined by LC-MS and NMR analysis, the MSI analysis was successful in detecting, mapping and identifying the molecular differences between the drug treated and vehicle control tissues directly in the tissue sections even without *a priori* knowledge and may therefore be a suitable standalone analysis in preclinical toxicology studies where identification and distribution of compounds is required.

### MALDI MSI Analysis of Drug Distributions

**Figure 3:**
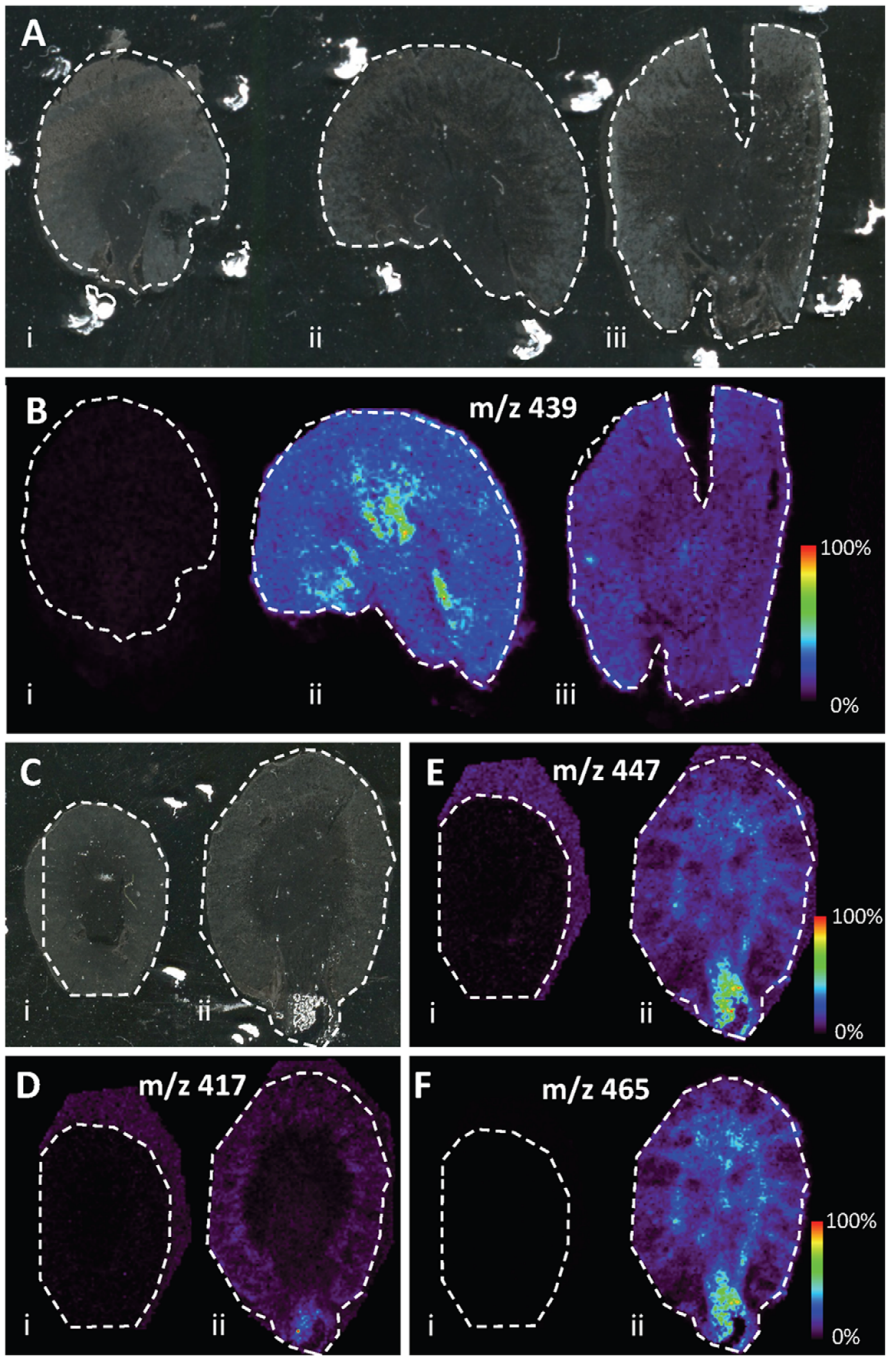
Ion distribution images of administered drug candidate compounds and some detected metabolites. A) Optical images of analyzed tissue sections. i; vehicle control, ii; compound 1 dosed animal tissue section with low crystal load, iii; compound 1 dosed animal tissue section with high density of crystals. B) Ion distribution of compound 1 (*m/z* 439) on the tissue sections in panel A. C) Optical images of analyzed tissue sections. i; vehicle control, ii; compound 2 dosed animal tissue section. D) Ion distribution image of compound 2 on the tissue sections in panel C. E) Ion distribution image of a compound 2 metabolite (doubly oxidized) on the tissue sections in panel C. F) Ion distribution image of compound 2 metabolite (triply oxidized) on the tissue sections in panel C. The samples were coated with sinapinic acid and analyzed in negative mode on the Synapt G2. The data was normalized by total ion count.

By using MSI, *t*he abundance and distribution of compound 1 (m/z 439) was measured across kidney sections from animals dosed with compound 1 that had a low occurrence of crystals (observed by histopathology) and from animals that showed a high occurrence of crystals. The two dosed samples were analyzed alongside a vehicle control kidney sections. Figure 3 shows that m/z 439 can clearly be detected in the kidney sections of compound 1 treated animals, with its distribution detected across both drug treated kidney sections (Figure 3B ii–iii, while no detection of m/z 439 was seen in the vehicle control tissue (Figure 3B i). However, the abundance of m/z 439 differs between the two tissue sections from two different animals given compound 1 (Figure 3B ii–iii). The sample with a higher occurrence of crystals (Figure 3A iii, 3B iii) has an abundance of the parent compound approximately 50% less than that seen detected in section with low crystal occurrence, suggesting that more of the parent drug may have been metabolized. The abundance and distribution of substance compound 2 (m/z 417) was also mapped by MALDI MSI. Figure 3D shows that the distribution of m/z 417 is only detected in the drug treated kidney section (ii) and not in the control (i) tissue. The signal observed outside the control kidney is a background artifact most likely due to different ionization efficiency on the glass slide compared to on tissue. This background signal is observed at the same intensity also outside the drug treated kidney section. Compound 2 can be seen to be at greatest abundance in the papillary region.

Finally in Figure 3E–F, MSI distributions of two drug metabolites of compound 2 are shown (LC-MS/MS confirmation data presented in Figure S2). While the parent compound was detected to localize predominantly in the papillary region, the two metabolites distributions can be seen to be correlated to the area of the kidney defined as both the medullary and papillary region. The histopathological examination of these regions revealed abundant depositions of crystalline material in predominantly the renal papilla. The crystalline material was often surrounded by an inflammatory reaction composed by neutrophilic granulocytes and macrophages. Such detections and distributions are examples of the how untargeted MSI analysis can be used to identify molecular changes in tissues, which will now be explored further.

### Untargeted Molecular Profiling and Imaging

In addition to the targeted MS and NMR analysis directed by the histopathological observations, an untargeted profiling analysis of the kidney tissue sections was performed with the aim of revealing any low molecular weight changes accompanying the observed kidney damage. Untargeted studies are often performed on tissue extracts, rather than whole tissues, as increased sensitivity can be obtained through use of LC separation prior to MS analysis. However, there is a risk of drug metabolite or endogenous compound loss or modification during the extraction process, as well as some possibility of sample contamination. Another benefit of MALDI MSI is that one can directly compare the distribution of the analytes found with the various morphological or toxicological patterns. Therefore, it is beneficial to analyze whole tissue directly. Parallel analysis of kidney tissues from dosed and vehicle control animals were performed with tissue extracts analyzed by LC-MS and intact sample analysis performed by NMR HRMAS and MALDI MSI. The HRMAS NMR analysis allows the acquisition of spectra from small pieces of intact tissue without any sample treatment and the MSI allowing the spatial distribution of compounds to be preserved.

**Figure 4:**
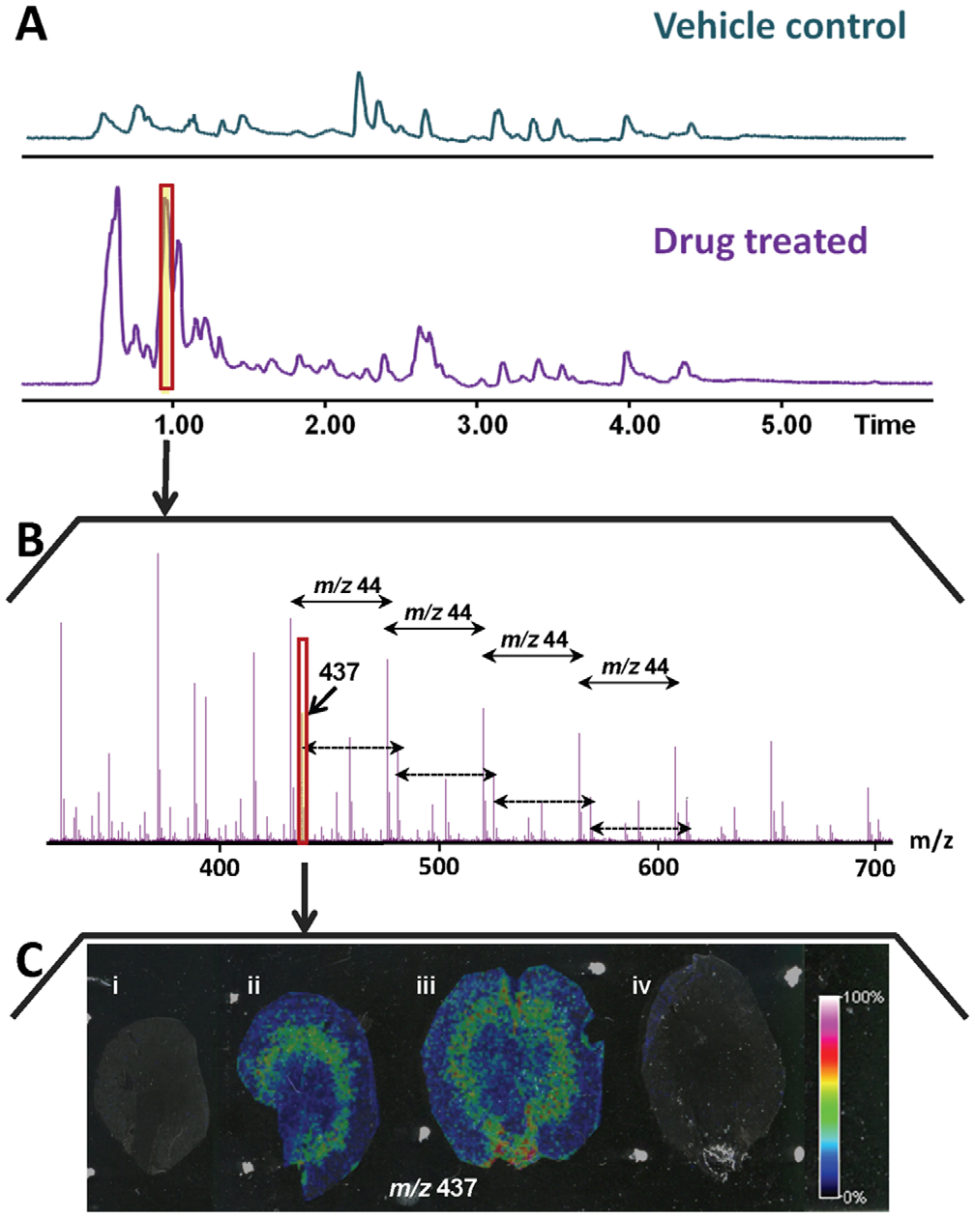
Results from an untargeted analysis of kidney tissue from drug treated animals and vehicle controls. A) Total ion chromatogram of kidney extract from the LC-MS analysis of vehicle control (upper) and animal dosed with compound 1 (low crystal load) (lower), run on an HILIC column. B) Mass spectrum from the peak marked in red on panel A, showing a PEG distribution with *m/z* 44 distance between peaks. C) The distribution of PEG on the tissue is represented by the ion distribution image of *m/z* 437. This distribution pattern is overlaid on the tissue sections from (i) vehicle control, (ii) animal dosed with compound 1 (low crystal load), (iii) animal dosed with compound 1 (high crystal load), and (iv) animal dosed with compound 2 (not formulated in PEG400). The MSI samples were coated with CHCA and analyzed in positive mode on the UltraFlex II. The data was normalized by total ion count.

Kidney tissue extracts from both treatment regimes were compared to vehicle control tissue extracts analyzed by LC-MS. A large number of additional peaks were detected in the drug treated samples compared to the vehicle control sample (Figure 4A). An examination of mass spectra showed that the majority of those peaks originated from different chain lengths of the PEG400 substance. A mass spectrum from the LC-MS analysis in positive ion mode can be seen in Figure 4B, with the typical PEG ladder distribution of increasing *m/z* 44 peaks. However, to confirm that PEG400 was present within the tissues and had not been introduced during sample processing, intact kidney sections were analyzed by HRMAS NMR and MALDI MSI. This HRMAS analysis was performed both to cross validate the MALDI MSI findings and to provide complementary information such as a more overall quantitative estimate on the findings than what can be obtained by MALDI MSI due to differences in ionization efficiency between compounds. The data from the HRMAS NMR-experiments was analyzed to determine the overall difference between dosed and control animals. The most significant results from this analysis, was a clear difference in a peak at δ 3.51 (Figure S3) that was present in tissue from all animals treated with compound 1 but in none of the two control rats. The NMR results indicated presence of long carbon chains, hence this peak was suggested to be PEG400 and addition of PEG400 (vehicle for compound 1) to the sample confirmed its identity.

MALDI MSI analysis was performed in positive mode following application of CHCA MALDI matrix and the same pattern of peaks were detected as found by the LC-MS analysis, showing repeating *m/z* 44 ladder, only in tissues that were treated with compound 1. The corresponding MSI distribution image for PEG400 is presented in Figure 4C. This distribution pattern suggests that PEG400 is accumulated in the area of the tissue that correlates to the previously described pathological findings. Significantly, PEG400 was used in formulation of compound 1 and the vehicle for the control animals. As no PEG400 was detected in vehicle control kidney sections we suggest that the toxicological damage has caused inability of the damaged kidneys to eliminate PEG400 through impaired excretion functionality. By a combination of analytical techniques, we are able to detect molecular distributions within tissue samples that could not be observed by histopathology which can provide an indication of the severity of tissue damage that can further aid our understanding of the toxicological events occurring.

### Conclusions

In the present study, we have shown how MALDI MSI, in combination with LC-MS and HRMAS NMR, can provide detailed information to a complex toxicological problem occurring in pre-clinical drug development. This type of information can be of great value if fed back to drug design programmes. Our study aims to demonstrate how multi-strand analysis can be combined to address and identify pharmaceutical research based toxicity events. Each of the techniques employed gave complementary information and cross validation of the data generated. NMR provided compound structural information and validation through the co-analysis of a compound standard but without spatial information. MALDI MSI provided detailed information of both spatial distribution and identification of compounds. While one methodology could have produced sufficient data, the results and conclusions drawn are reinforced by those generated by the alternative techniques.

The identity of crystal deposits occurring following administration two candidate drugs that caused kidney damage have successfully been determined by the combination of MALDI MSI analysis with LC-MS and NMR analysis of crystals isolated by manual dissection. Taken together the results from the present study suggest that that the metabolite bisulphonamide is responsible for the kidney toxicity previously observed following exposure to compound 1 and compound 2. It could also be concluded that when PEG400 is administered as a vehicle together with one of the drugs shown to cause tissue damage (e.g. compound 1), it is depositing and accumulating in the damaged kidney tissue indicating that the damage might be preventing clearance of the vehicle. This accumulation was not seen in control animals administered with PEG400 vehicle only. Therefore, the results show that by using MALDI MSI we are able to identify molecular changes correlating with pathology associated with kidney damage. Overall, we show that MSI is very useful for the detection and visualization of small molecules and therefore a suitable technology platform to implement in early drug development and discovery processes.

## Supporting Information

Figure S1 **MS/MS of crystals directly from tissue sections.** A) Scanned kidney tissue sections from a control (left) and a dosed animal (right). Areas analyzed by MALDI MSI are marked on the tissue in white. Bisulphonamide standard is deposited on the control tissue (0.2 µL of 12 ng/mL) at four different locations (circled in red). B) Extracted ion distribution image of *m/z* 171 on the analyzed areas in panel A. C) MS/MS spectrum of bisulphonamide standard on control tissue. D) MS/MS spectrum of bisulphonamide on tissue from an animal dosed with compound 2.
(TIFF)

Figure S2 **MS/MS analysis of compound 2 and its metabolites.** All three compounds produce common and specific fragments, which originates from the bisulphonamide part of the structure. A) MS/MS of compound 2 detected at *m/z* 417 with structure presented in panel 1. B) MS/MS of metabolite detected at *m/z* 447 with suggested structure in panel 2. C) MS/MS of metabolite detected at *m/z* 465 with suggested structure in panel 3.
(TIF)

Figure S3 **NMR spectra from kidney tissue extracts.** The PEG400 peak is found at δ 3.5.
(TIF)

Text S1 More detailed information about the experimental procedures.
(DOCX)
