# Supplementary figures and images for: In Situ Mass Spectrometry Imaging and Ex Vivo Characterization of Renal Crystalline Deposits Induced in Multiple Preclinical Drug Toxicology Studies
Source: PLoS One. 2012 Oct 23;7(10):e47353. doi: 10.1371/journal.pone.0047353 (PMC3479109; doi:10.1371/journal.pone.0047353)

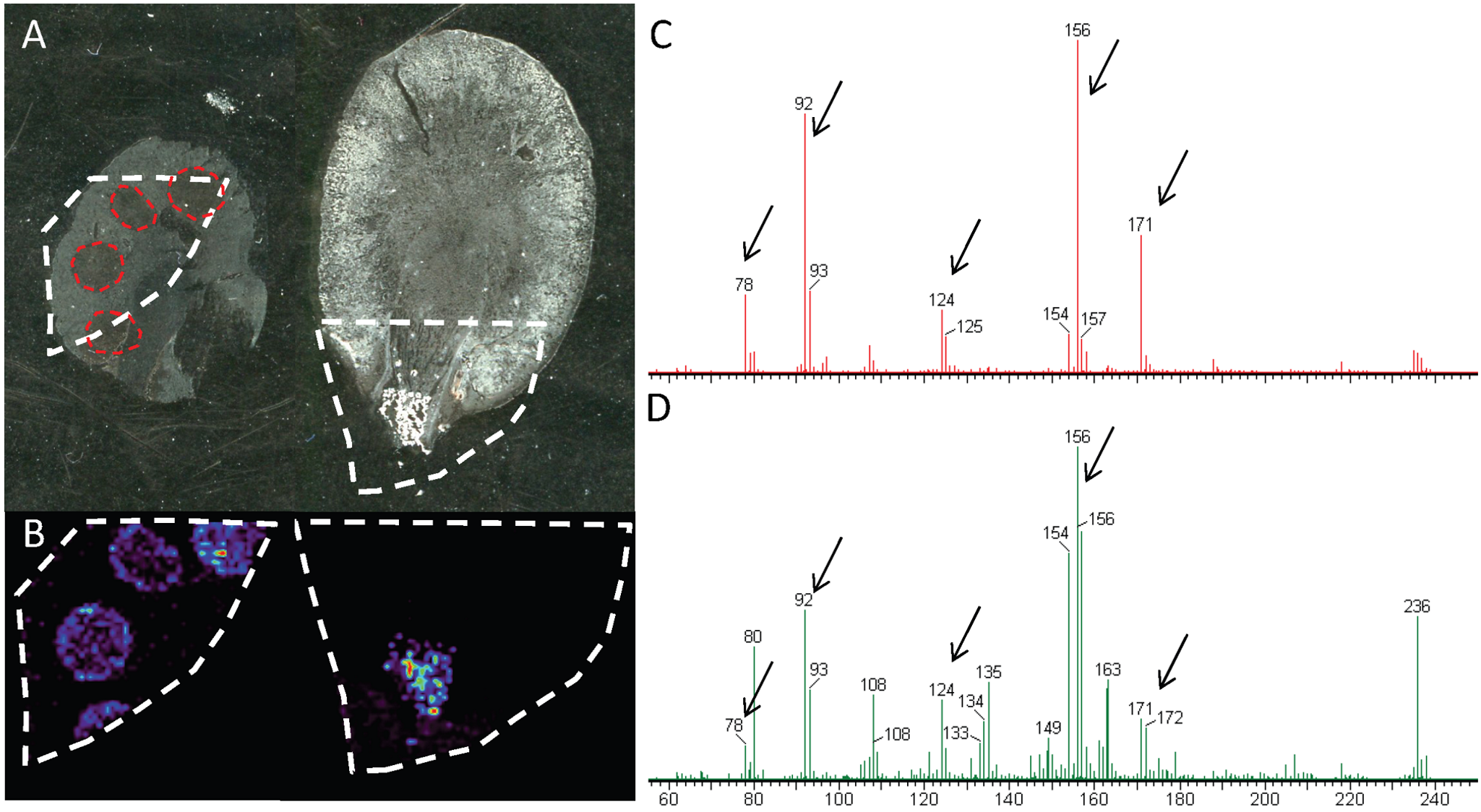

Supplement: Figure S1 — MS/MS of crystals directly from tissue sections. A) Scanned kidney tissue sections from a control (left) and a dosed animal (right). Areas analyzed by MALDI MSI are marked on the tissue in white. Bisulphonamide standard is deposited on the control tissue (0.2 µL of 12 ng/mL) at four different locations (circled in red). B) Extracted ion distribution image of m/z 171 on the analyzed areas in panel A. C) MS/MS spectrum of bisulphonamide standard on control tissue. D) MS/MS spectrum of bisulphonamide on tissue from an animal dosed with compound 2. (TIFF) [file pone.0047353.s001.tif]

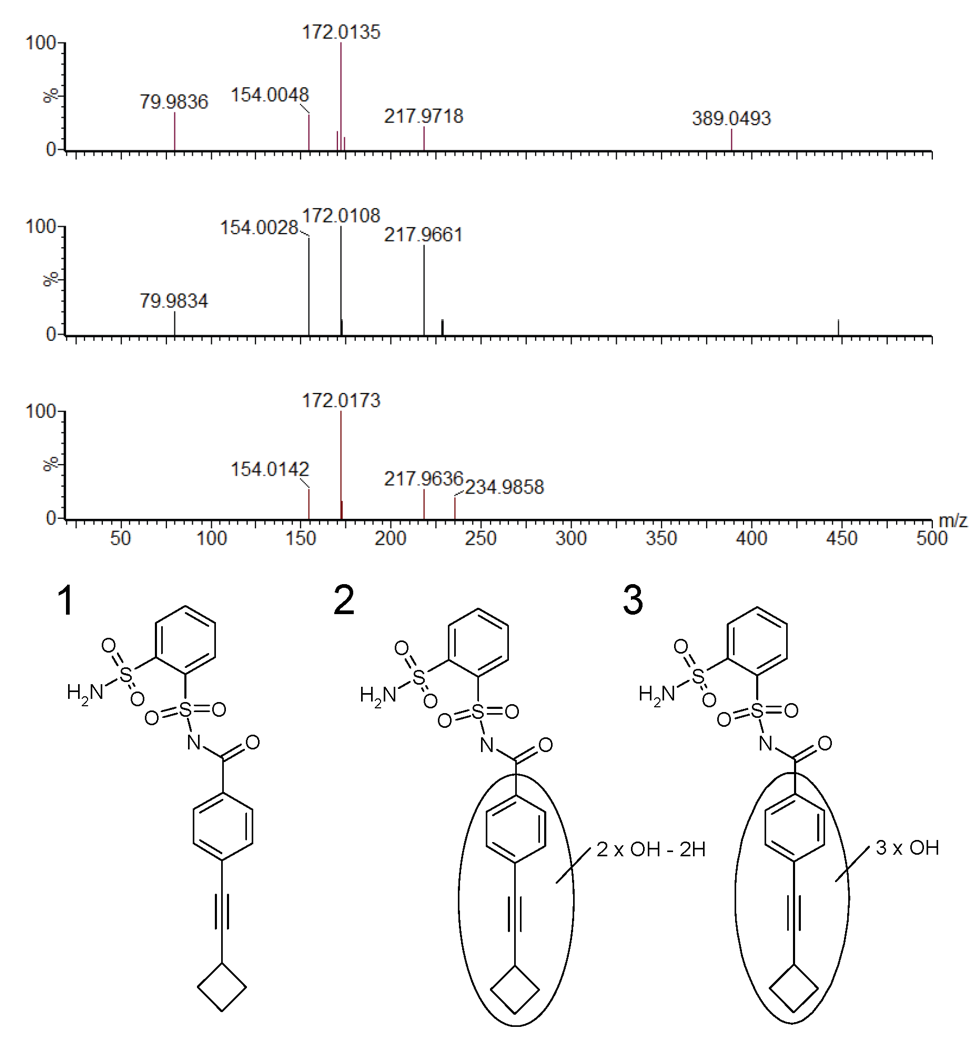

Supplement: Figure S2 — MS/MS analysis of compound 2 and its metabolites. All three compounds produce common and specific fragments, which originates from the bisulphonamide part of the structure. A) MS/MS of compound 2 detected at m/z 417 with structure presented in panel 1. B) MS/MS of metabolite detected at m/z 447 with suggested structure in panel 2. C) MS/MS of metabolite detected at m/z 465 with suggested structure in panel 3. (TIF) [file pone.0047353.s002.tif]

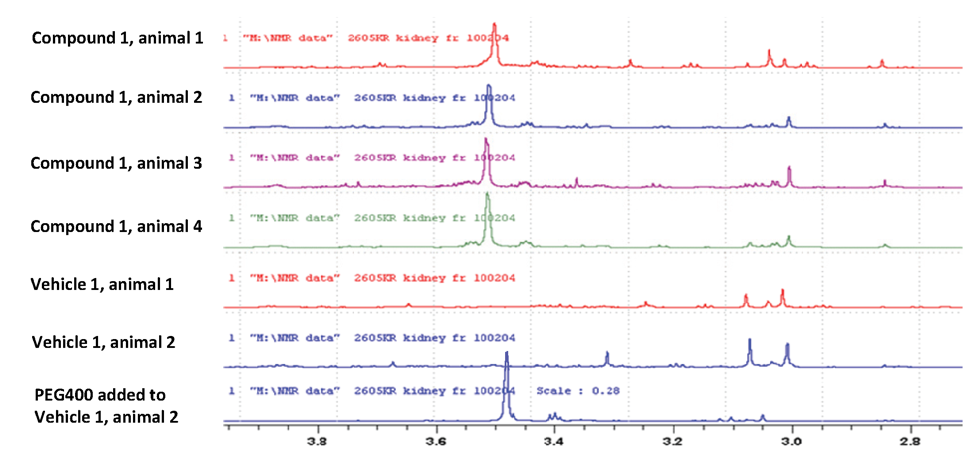

Supplement: Figure S3 — NMR spectra from kidney tissue extracts. The PEG400 peak is found at δ 3.5. (TIF) [file pone.0047353.s003.tif]
