# Supplementary material for: In Situ Mass Spectrometry Imaging and Ex Vivo Characterization of Renal Crystalline Deposits Induced in Multiple Preclinical Drug Toxicology Studies
Source: PLoS One. 2012 Oct 23;7(10):e47353. doi: 10.1371/journal.pone.0047353 (PMC3479109; doi:10.1371/journal.pone.0047353)
Supplement: Text S1 — More detailed information about the experimental procedures. (DOCX) [file pone.0047353.s004.docx]

SI -Supplementary Information

Experimental procedures

Animals

Animals were multiple-housed in transparent plastic cages with aspen wood chips as bedding material. Plastic tunnels and aspen chew blocks were provided. Temperature and relative humidity were 17-23°C and 40-70%, respectively.   The animal room was illuminated by artificial light from fluorescent tubes on a 12 hour light/dark cycle. Animals had free access to tap water and RM1 (E) SQC pelleted diet (Special Diets Services Ltd, England). The rats were acclimatized for at least 10 days prior to the experiment.

Tissue samples

In all animals the kidneys were cross sectioned into two halves of equal size. The kidney halves intended for histological examination were fixed in a 4% buffered formalin solution (Solveco AB, Sweden), embedded in paraffin, sectioned at 4 µm and stained with hematoxylin and eosin. The other halves were snap frozen in a mixture of isopentane and dry-ice and were then kept at -80˚C until analysis. These snap frozen kidneys were sectioned for MALDI MSI, NMR HRMAS, and kidney extract preparation. For the MALDI MSI analysis, 8 µm thick kidney sections were cut and thaw-mounted onto indium thin oxide (ITO) coated MALDI target slides (Bruker Daltonics, Bremen, Germany Cat. #237001). Every fifth section was mounted on a superfrost plus slide for histology examination. Water was used as the mounting media to avoid any contamination from embedding media normally used. Tissue sections were taken at approximately the same level in the kidney and sections from dosed animals were mounted adjacent to vehicle controls to minimize variability due to differences in matrix coating or time of analysis. Mounted tissue sections were transported on dry-ice and stored in -80°C until analysis. For the HRMAS analysis and kidney extracts, 50 µm thick sections were cut. Four sections were collected 6 times with two 8 µm slices for histopathology analyzes in between. The sections were placed in the HRMAS-rotor and 25-50 µL of DMSO-d6 was added to the samples. All sections were cut using a cryostat microtome (Leica CM3050S, Leica Microsystems AB, Germany).

**Histology**

Tissue section from formalin-fixed and paraffin embedded materials and fresh frozen tissue sections on MALDI glass targets (post MALDI MSI analysis) were H&E stained according to the following protocol. Slides are washed four times for five min in xylene before rehydration in absolute ethanol and 95 % ethanol two times five min each, in 70 % ethanol one min and the same time in water. Subsequently, the sections are stained in Gills II hematoxylin for 8 min, rinsed in distilled water, transferred to 70% ethanol and differentiate and blue cell nuclei in 70%  ethanol with some drops ammonium hydroxide, 3-5 times agitation and dehydrated in 70 % ethanol, 95 % ethanol and 100 % ethanol, then cleared twice in xylene for 2 min and mounted with pertex. After staining and mounting, all tissue sections were scanned in an Aperio ScanScope (Aperio Technologies Inc., CA, USA) at 20 times magnification and the scanned images from MALDI glass targets were overlaid with the MALDI MS images.

Crystal isolation

Crystals were manually dissected from kidney tissue. Consecutive tissue sections were stained with hematoxylin and eosin and used for reference during the manual dissection. The tissue sections were taken from -80°C and dried in room temperature in glass cuvettes for approximately 5 minutes. Dissection was performed with a needle and the samples were then dried in vacuum in 2 mm eppendorf tubes for 10 minutes to remove water. The unstained sections were observed in the microscope and crystals were located as they reflect light under the microscope. The crystals obtained were dissolved in 10 µL NMR solvent (DMSO-d6), vortexed, and transferred to a 1 mm NMR tube prior to analysis. 1 µL of this sample was used for LC-MS analysis and the remaining sample was used for NMR analysis. In reversed phase chromatography LC-MS, DMSO is a solvent with high eluting strength. Therefore 1 µL of this solvent was diluted with 30 µL of acetonitrile and only 1 µL of the diluted sample was injected on the chromatography column.

**MALDI MSI analysis**

*Matrix Application*

Three different MALDI matrices, alpha-cyano-4-hydroxycinnamic acid (CHCA, 10 mg/mL), sinapinic acid (SA, 10 mg/mL), and 2,5-dihydroxybenzoic acid (DHB, 60 mg/mL) in 50:50:0.1 ACN/H_2_O/TFA were mixed with drug standards (compound 1, compound 2 and bisulphonamide) to determine optimum ionization conditions. 0.5 µL of drug mixed with matrix solution was spotted on to either MALDI target plate or control kidney tissue (12 ng/mL, 1.2 ng/mL). Samples were analyzed both in positive and negative mode on the UltraFlex II (Bruker Daltonics). For the initial analyzes of the manually deposited spots, spectra consisting of 1000 laser shots were acquired as 5 x 200 shots and data was collected between *m/z* 140-1440 Da.

Prior to matrix application, drug standards (compound 1, bisulphonamide (12 ng/mL, and 1.2 ng/mL in 50:50 ACN/H_2_O) were manually spotted onto control kidney tissue. Optical images of the tissue sections were taken using a standard flat bed scanner (Epson Perfection V500 photo, Seiko Epson Corp., Japan). For analysis in negative mode, the kidney tissue sections were coated with SA matrix solution (7.5 mg/mL in 70:30:0.1 ACN/H_2_O/TFA), and for positive mode CHCA was used (5 mg/mL in 50:50:0.1 ACN/H_2_O/TFA). The matrix was applied using an automatic spraying device (Image prep, Bruker Daltonics). Briefly, a matrix aerosol is created by vibrational vaporization under controlled conditions with all droplet diameters ≤ 50 µm and an average droplet size of ~20 µm. A customized predefined method was used for matrix application using the ImagePrep. The thickness of the matrix layer was monitored by the output from the optical sensor and four cycles of equal amount of matrix were applied while turning the slide 180 degrees between each cycle. Total thickness of matrix layer was corresponding to 1.0-1.05 V. The coated tissue sections were dried in the desiccator for about 20 min before MSI analysis.

*MALDI MSI acquisition*

MALDI MSI experiments were carried out using the UltraFlexII MALDI-TOF/TOF MS (Bruker Daltonics) equipped with a solid-state Smartbeam laser operating at 200 Hz or the Synapt G2 HDMS system (MALDI Q-TOF MS, Waters Corp., U.K.) equipped with an Nd:YAG laser operating at 1 kHz.

Using the Ultraflex II, the laser spot size was set at medium focus (laser spot diameter of ~50 µm) and after optimization of laser power intensity in the beginning of each run, the laser power was fixed for the rest of the experiment. The instrument was calibrated using a standard peptide mix (Bruker Daltonics) with addition of the matrix cluster peaks of SA (negative mode; *m/z* 223.06 and *m/z* 447.13, positive mode; *m/z* 225.08 and *m/z* 449.14). In positive mode, data was acquired in the range of *m/z* 140-1680 at a resolution of 150µm, collecting 300 shots/spot. Ion deflection up to 120 Da was activated and the sampling rate was 1.0 GS/s. FlexImaging 2.0 (Bruker Daltonics) was used for setting up the imaging experiment. The Ultraflex II can produce data with a mass resolution up to 20,000 with mass accuracy below 5 ppm. A single spectrum from tissue of 300 shots typically generated a mass resolution of 2,000-5,000 with a mass accuracy around 10 ppm following external calibration in the mass range < 500 Da.

For analysis on the Synapt G2 HDMS system, the instrument was calibrated in negative mode with CsI (2 mg/mL in 50:50 isopropanol:water). The data was acquired in negative mode using sensitivity mode in the range of m/z 150-600 with 330 laser shots per spot at a laser repetition rate of 1 kHz. The area selected for imaging was defined using the MALDI Imaging Pattern Creator (Waters Corp.). Spatial resolution of 100 µm was used. Sample plate voltage was 0 and extraction voltage 10. Collision voltage of 30 eV was used for the MS/MS experiments. The Synapt G2 HDMS can produce data with a mass resolution up to 40,000 with mass accuracy below 1 ppm. A single spectrum from tissue of 330 shots has typically generated a mass resolution of 10,000-15,000 with a mass accuracy around 10 ppm following external calibration in the mass range <500 Da.

*MALDI MSI data analysis*

The data acquired on the Ultraflex II was analyzed and normalized using FlexImaging 2.0. The tissue section from each individual animal was defined as a region of interest using the optical scanned image. Ion distribution images of masses where average abundance was different between tissues from control and dosed animals were mapped with a mass filter precision of ± 0.1 Da.

Following acquisition on the G2 Synapt, raw data was converted into Analyze file format using MALDI imaging converter (Waters Corporation, Manchester, UK) and normalized by total ion current using an in-house written script. Subsequent data analysis was performed using BioMap (Novartis, Switzerland) and Origin 8 (Origin Lab, USA). Ion distribution images were extracted using a mass filter of approximately ± 0.015 Da depending on peak shape. The mass filter was kept constant for a specific mass on different tissues.

NMR analyses

The NMR spectroscopic measurements were made on a Bruker 600 MHz instrument (Bruker BioSpin, Rheinstetten, Germany) equipped with a sample changer. An HRMAS probe was used for the kidney tissue and a 1 mm TXI probe was used to analyze the DMSO-d6 phase from kidney tissue after separation of the tissue and DMSO-d6 dissolved samples of isolated crystals.

Reference samples of compound 1 and bisulphonamide were dissolved in DMSO-d6 before analysis. The vehicle PEG400 was delivered as an aqueous solutions (20% (v/v) Polyethylene Glycol (PEG400) in 0.2 M Meglumine) and were run like that with TSP added as reference in NMR experiments.

Kidney tissue preparation and extraction

The kidneys used for extraction experiments and HRMAS were cryo sectioned. 4 slices of 50 µm where collected 6 times with two 8 µm slices for pathology analyses in between. The sections were placed in the HRMAS-rotor before 25-50 µL of DMSO-d6 was added to the samples. After NMR analysis, tissue and DMSO-d6 phase were separated into different tubes by centrifugation (1500 rpm for 5 min, room temperature, Hermle Z513, Hermle Labortechnik GmbH, Germany).

*Kidney tissue NMR HRMAS analysis*

^1^H-NMR HRMAS spectra of kidney tissue were acquired using a Bruker standard cpmg presat puls program, [relaxation delay-90°-refocus delay -acquire-FID]. The residual water resonances were suppressed by presaturation during the relaxation and refocusing delay with a total T_2_ relaxation delay of 80 ms. 512 scans were collected into 64k time domain data points and processed using a exponential window function with a line broadening factor of 0.3 Hz and zero-filled to yield a real spectrum of 64K data points in the frequency domain after Fourier transformation.

The resulting spectra were phase and baseline corrected manually and referenced to DMSO at δ 2.50.

*NMR analysis of crystal sample*

^1^H-NMR spectra of crystal samples dissolved in DMSO-d6 were acquired using 1D-experiments (cpmgpr, noesygppr1d and zgesgp Bruker pulse sequences). 64K data points were collected using 2048 scans. All measurements were performed at 300 K and water suppression was achieved either with either a presaturation pulse on the water frequency or with the excitation sculpting scheme. Sampled data were zero-filled to 256K points and apodized using an exponentional function with a line broadening factor of 0.3 Hz prior to Fourier transformation. All data acquisition and processing was done with Topspin2.1 (Bruker).

LC-MS analysis

*Sample preparation*

LC-MS samples were prepared by dilution from the DMSO-d6 phase remaining from the NMR experiment. 1) For HSS analyses of tissue extracts; 1 µL DMSO-d6 phase was diluted with 70 µL water. 2) For HILIC analyses of tissue extracts; 2 µL extract DMSO-d6 phase was diluted with 100 µL acetonitrile. 3) For crystal sample analyses; 1 µL DMSO-d6 phase was diluted with 30 µL acetonitrile for all analyses.

The LC-MS instrument used was a UPLC/Q-TOF MS (Waters Corp., U.K.). Kidney extracts were analyzed in both positive and negative ionization modes using two different chromatographic systems; 1) A HSS T3, 2.1x100 mm, 1.8 µm column, with a flow rate of 0.6 mL/min. Mobile phase A was 0.1% formic acid in water and mobile phase B was 0.1% formic acid in acetonitrile. The gradient was 1-20% B 0-5.5 min, 20-90% B 5.5-9 min, 90% B 9-13 min. 2) A BEH Amide 2.1x100 mm, 1.7 µm column, with a flow rate of 0.3 mL/min. Mobile phase A acetonitrile and mobile phase B was 5 mM formic acid in water. The gradient was 10-50% B 0-5 min. A volume of 5 µL of sample was injected for all analyses of extracts. For analysis of dissected crystals the HSS method was used in negative ion mode and 1 µL was injected from the sample. The mass spectrometer was operated in full scan mode (m/z 50-1000), with the collision cell held at 5 V in MS mode. MS/MS experiments were performed by at collision energies of 5V, 10 eV and 20 eV respectively, to obtain MS/MS spectra at different energies.

For profiling analysis of kidney extracts, an in-house designed software, TracMass, was used to extract the most distinct differences between the samples.
